# C1q/TNF‐related protein‐9 promotes macrophage polarization and improves cardiac dysfunction after myocardial infarction

**DOI:** 10.1002/jcp.28513

**Published:** 2019-04-05

**Authors:** Mingxin Liu, Lin Yin, Wei Li, Juan Hu, Huibo Wang, Bingjie Ye, Yanhong Tang, Congxin Huang

**Affiliations:** ^1^ Department of Cardiology Renmin Hospital of Wuhan University Wuhan Hubei China; ^2^ Cardiovascular Research Institute of Wuhan University Wuhan Hubei China; ^3^ Hubei Key Laboratory of Cardiology Wuhan Hubei China; ^4^ Department of Cardiovascular Medicine Xiangya Hospital, Central South University Changsha Hunan People's Republic of China; ^5^ National Clinical Research Center for Geriatric Disorders Xiangya Hospital, Central South University Changsha Hunan People's Republic of China; ^6^ Institute of Hypertension Central South University Changsha Hunan China

**Keywords:** cardiac function, CTRP9, inflammation, macrophage polarization, myocardial infarction

## Abstract

The timely regulation of inflammatory M1 macrophage polarization toward regenerative M2 macrophages suggests the possibility of immunotherapy after myocardial infarction (MI). C1q/TNF‐related protein‐9 (CTRP9) has anti‐inflammatory effects and can ameliorate heart function in mice after long‐term myocardial infarction. The role of CTRP9 in macrophage polarization remains completely unclear. This study determined whether CTRP9 can preserve post‐MI early cardiac function through the regulation of macrophage polarization. In the present study, an adenovirus‐delivered CTRP9 supplement promoted macrophage polarization at Day 3 post MI and improved cardiac function at Day 7 post MI. Pretreatment with gCTRP9 promoted the M1 to M2 polarization transition and attenuated inflammation after lipopolysaccharide + interferon‐γ stimulation; the effects were partly abrogated by the adenosine monophosphate kinase (AMPK) inhibitor compound C and were obviously reinforced by pyrrolidine dithiocarbamate, a nuclear factor‐κB (NF‐κB) inhibitor. Meanwhile, CTPR9 markedly reduced the expression of toll‐like receptor 4 (TLR4), myeloid differentiation factor 88 (MyD88), and NF‐κB p65 phosphorylation by promoting AMPK phosphorylation in vivo and in vitro. Moreover, the competitive binding of gCTRP9 and LPS to the myeloid differentiation protein 2 (MD2)/TLR4 complex was associated with direct binding to MD2, thereby inhibiting the downstream signaling molecule MyD88. Taken together, we demonstrated that CTRP9 improved post‐MI early cardiac function, at least in part, by modulating M1/M2 macrophage polarization, largely via the TLR4/MD2/MyD88 and AMPK‐NF‐κB pathways.

## 1 INTRODUCTION

Inflammation subsidence is important for improving tissue injury and cardiac function after myocardial infarction (MI) and requires the concerted action of macrophages. Peripheral monocytes/macrophages and cardiac‐resident macrophages migrate to the infarct and border region, differentiate into M1 macrophages, and exacerbate cardiac function. Thereafter, entering the inflammation resolution stage, the dominant macrophage populations of myocardial tissues become reparative M2 macrophages that accelerate cardiac repair (Gombozhapova et al., 2017; Tokutome et al., 2018). Inflammation from M1 macrophages is an essential component of early ventricle remodeling, and prolonged inflammation may damage the physiology of the left ventricle (LV) by raising LV expansion and redundant scar transformation (Ben‐Mordechai et al., 2015; Gombozhapova et al., 2017). Many studies have emphasized the therapeutic potential of pharmacological regulators in the treatment of myocardial injury through the modulation of macrophage polarization (Cheng & Rong, 2018; Shirakawa et al., 2018). Regarding the features of phenotypic dynamics after MI, M1 macrophages predominate early at 1–3 days and peak on Day 3, whereas M2 macrophages gradually predominate during inflammation regression after 5 days (Christia et al., 2013; Mouton et al., 2018; Yan et al., 2013). Earlier phenotype transformation of M1 to M2 macrophages has resulted in prominent improvements in MI wound healing and cardiac function (Choo et al., 2017). Hence, positively transforming to the M2 phenotype at 3 days is a therapeutic key to reduce adverse LV remodeling after MI.

It has been well‐known that macrophages are also functionally polarized into M1 and M2 cells in response to infection with microorganisms and host mediators. Whereas lipopolysaccharide (LPS) and/or interferon‐γ (IFN‐γ) induce M1 macrophage generation, stimulation of macrophages with interleukin (IL)‐4 or IL‐13 induces M2 macrophages (Biswas, Chittezhath, Shalova & Lim, 2012; Vats et al., 2006). Macrophage phenotypes are defined by the expression patterns of specific biomarkers, inducible nitric oxide synthase (iNOS) and CD86 are well‐recognized as key M1 biomarkers (Gong, Zhuo & Ma, 2017; Martinez, Helming & Gordon, 2009). M1 macrophages express abundant proinflammatory mediators, including tumor necrosis factor (TNF)‐α, IL‐1 and IL‐6, which mediate tissue damage and impair tissue regeneration and wound healing, whereas the M2 phenotype expresses molecules including arginase1 (Arg1), CD163, and IL‐10, which protect against tissue damage, promote wound healing, and possess anti‐inflammatory properties (Gordon & Martinez, 2010; Moore, Sheedy & Fisher, 2013). The mechanisms that control the balance between M1 macrophages and M2 macrophages are particularly important. Many key transcription factors are involved in macrophage polarization, such as interferon‐regulatory factor (IRF5), activator protein1 (AP1), peroxisome proliferator‐activated receptor‐γ (PPAR‐γ; Y. C. Liu, Zou, Chai & Yao, 2014), nuclear factor‐κB (NF‐κB; Dai et al., 2015; Oeckinghaus, Hayden & Ghosh, 2011), and adenosine monophosphate kinase (AMPK; Sag, Carling, Stout & Suttles, 2008; Weng & Schuppan, 2013), which interact with each other and regulate macrophages to acquire a certain phenotype in various inflammatory diseases. Toll‐like receptor 4 (TLR4), a pattern recognition receptor, plays a pivotal role in inflammation and the immune system, whereas the key transcription factors AP1, IRF5, and NF‐κB are the downstream signaling molecules of TLR4 in the myeloid differentiation factor 88 (MyD88)‐dependent pathway and exert proinflammatory effects (Y. Yang et al., 2016).

The C1qTNF‐related proteins (CTRPs) are a recently discovered and highly conserved family of adiponectin (APN) paralogs. Among CTRPs, CTRP9 is highly expressed in heart and adipose tissue (Appari et al., 2017). The globular domain isoform of CTRP9 (gCTRP9) is generated via proteolytic cleavage of full‐length CTRP9 (fCTRP9) and is the main active isoform (Y. Yuan et al., 2015). Studies have shown that CTRP9 has anti‐inflammatory effect via AMPK activation (Kambara et al., 2015; Zhang et al., 2016) and can attenuate adverse cardiac remodeling in long‐term MI, largely via a protein kinase A (PKA)‐dependent pathway (Sun et al., 2013). Simultaneously, we consider that cardiac CTRP9 expression might be regulated by PPAR‐γ (Su et al., 2013), and CTRP9 prevents activation of the TLR4‐MyD88‐p65 pathway by activating AMPK to inhibit the cholesterol‐induced vascular smooth muscle cell (VSMC) phenotype switch (Q. Liu et al., 2017). Conversely, we have known that the signaling molecules TLR4, AMPK, NF‐κB, and PPAR‐γ, which are associated with CTRP9, are involved in macrophage polarization. Given this evidence, whether CTRP9 can regulate cardiac function in the early stage post MI by affecting macrophage polarization and the specific mechanism remain largely unknown.

In the present study, we found that overexpression of CTRP9 in an MI model can enhance M2 macrophage polarization and improve cardiac function in the early stage post MI. gCTRP9 can reduce the inflammatory response by promoting the M1 to M2 macrophage transition in vitro, which was mechanistically associated with the TLR4/MD2/MyD88 and AMPK‐NF‐κB signaling pathways. In general, we provided evidence that CTRP9 acts as a critical regulator of cardiac function and macrophage polarization through suppression of the NF‐κB signaling pathway.

## 2 MATERIALS AND METHODS

### 2.1 Adenoviral vector construction

A recombinant adenovirus (Ad) encoding full‐length rat CTRP9 (Ad‐CTRP9; PubMed No. NM_001191891) or green fluorescent protein (Ad‐GFP) for the control virus was constructed and amplified. After confirming the sequence, we purified the recombinant virus and quantified the final plaque‐forming units (pfu; Genechem Co., Ltd., Shanghai, China).

Knockdown of CTRP9 was carried out using adenoviral vectors carrying CTRP9 small hairpin RNAs (shRNAs), which were generated by Hanbio (Shanghai, China). Scrambled shRNA was used as the control. The source of shCTRP9 is described as follows: The three target sequences of the CTRP9 gene for RNAi were CGAATTCAACCATTATGATACAGCA, named CTRP9‐shRNA1; CAGGAGGAGAGAGGTTCAATGGCTT, named CTRP9‐shRNA‐2; and GGAGAGAGGTTCAATGGCTTGTTTG, named CTRP9‐shRNA3. The negative control‐shRNA sequence was TTCTCCGAAC‐GTGTCACGTAA. Cells were transfected with these shRNAs, and messenger RNA (mRNA) expression of CTRP9 was detected by reverse‐transcription quantitative polymerase chain reaction (RT‐qPCR). CTRP9‐shRNA3 was the most efficient construct for knocking down CTRP9 expression.

### 2.2 Experimental protocols in vivo and cardiac function

All in vivo experiments were performed with adult male Sprague–Dawley (SD) rats weighing 180–220 g and were approved by the Animal Care and Use Committee of the Renmin Hospital of Wuhan University. This study conforms to the *Guide for the Care and Use of Laboratory Animals* published by the US National Institutes of Health (8th edition; NRC 2011). Rats were anesthetized with an intraperitoneal injection of 3% pentobarbital sodium (40 mg/kg). Then, MI was induced via left anterior descending coronary artery ligation as previously described (M. J. Yuan et al., 2016). For all procedures, samples were randomized and analyzed in a blinded manner. Animals were randomly divided into four groups: Sham, MI, Ad‐GFP + MI, and Ad‐CTRP9 + MI. Then, 3 × 10^9^ pfu of Ad‐CTRP9 or Ad‐GFP was injected into the jugular vein of each rat 5 days before MI. The Ad‐CTRP9 dose was based on previous research (Kambara et al., 2015), according to the conversion of the dose/body weight from mice. Nonsurviving rats underwent an autopsy and cardiac rupture was confirmed by the presence of coagulated blood in the thoracic cavity or observation of the crevasse on the ventricle. Echocardiography was performed as previously described at 7 days (per group, successively, *n* = 7, 6, 6, and 7; M. Yang, Xiong, Zou, Wang & Huang, 2018). After 3 or 7 days, rats were killed by intracardiac injection of KCl to induce diastolic arrest of cardiac activity. At the end of the study, all hearts were used to perform PCR, western blot analysis and enzyme‐linked immunosorbent assay (ELISA) at the peri‐infarct area (>2 mm outside the infarct).

### 2.3 Cell culture and treatment

Rat peritoneal macrophages were acquired from Procell Life Science & Technology Co, Ltd (Procell, Wuhan, China). We briefly describe the preparation process for peritoneal macrophages. Peritoneal macrophages were collected through the intraperitoneal injection of 3% thioglycollate medium (15 ml) and centrifugation of the extracted intraperitoneal internal liquid. CDl1b^+^ cells detected by flow cytometry were greater than 90%. Cultured macrophages were stimulated with 100 ng/ml LPS (L3129; Sigma‐Aldrich, St. Louis, MO) + 20 ng/ml IFN‐γ (400‐20‐20; Peprotech, Rocky Hill, NJ) or with 20 ng/ml recombinant rat IL‐4 (400‐04‐20; Peprotech; Monsalve et al., 2006; Vats et al., 2006) for 24 hr in the presence or absence of gCTRP9 (1, 3, and 5 μg/ml; 00081‐01‐100; Aviscera Bioscience, Santa Clara, CA; Zhang et al., 2016). Supernatants were collected for cytokine analysis by ELISA, and the cells were harvested for PCR, western blot or flow cytometry analysis. Otherwise, macrophage cells treated with gCTRP9 (3 μg/ml) at different time points (0, 15, 30, 60, and 120 min) were used for western blot analysis. In other experiments, the cells were preincubated with the AMPK inhibitor compound C (HY‐13418A; 10 mol/L; MCE, Monmouth Junction, NJ), the specific NF‐κB pathway inhibitor pyrrolidine dithiocarbamate (PDTC; HY‐18738; 100 μM; MCE), or vehicle (dimethyl sulfoxide) for 2 hr before CTRP9 treatment. To verify the expression of CTRP9 in different cells, we isolated and cultured rat primary cells from four different tissues, including cardiomyocytes, adipose, endotheliocytes, and peritoneal macrophages. To knock down CTRP9, macrophage cells were infected with Ad‐shCTRP9 (MOI = 150) or Ad‐shRNA for 24 hr. The cells were harvested for RNA and protein isolation.

### 2.4 Quantitative PCR analysis

Total RNA was extracted from cells by TRIzol reagent (15596‐026; Invitrogen, Carlsbad, CA), and the conversion of mRNA into cDNA was performed using an RT kit with genomic DNA Eraser (RR047A; TaKaRa, Kusatsu, Shiga, Japan). RT‐qPCR was performed using SYBR Premix Ex *Taq*II (RR420A; TaKaRa) in a StepOne™ Real‐Time PCR Detection System (Invitrogen Life Technologies). The primer sequences used are listed in Table 1. Changes in RNA transcripts were calculated by the $2^{-\Delta \Delta C_{t}}$ method, and gene expression levels were normalized to the expression of glyceraldehyde 3‐phosphate dehydrogenase.

**Table 1:** Information of Primers for RT‐qPCR

Gene name	Forward sequence (5′–3′)	Reverse sequence (5′–3′)
Rat‐Arg1	ATTGGCAAAGTGATGGAAGAGAC	CAAGACAAGGTCAACGCCAC
Rat‐CD163	AGCTAGGATGCCCAACTTTGAT	TCTTCCTGAGCATCGGTTGTC
Rat‐iNOS	AGCATCCACGCCAAGAACG	GTCTGGTTGCCTGGGAAAAT
Rat‐CD86	TAAGCAAGGATACCCGAAACC	CCGGGAATGGAAGAGATAGG
Rat‐CTRP9	GGCTTCTACTGGTTATGGACGC	GGAGCCTGGATCACCTTTGAT
Rat‐MMP‐2	TCCAATGATGACATCAAGGGG	GTCCGCCAAATAAACCGATC
Rat‐GAPDH	CGCTAACATCAAATGGGGTG	TTGCTGACAATCTTGAGGGAG

*Note*. Arg1: arginase1; CTRP9: C1q/TNF‐related protein‐9; GAPDH: glyceraldehyde 3‐phosphate dehydrogenase; iNOS: inducible nitric oxide synthase; MMP: matrix metalloproteinase.

### 2.5 Western blot

Protein extracts were subjected to sodium dodecyl sulfate‐polyacrylamide gel electrophoresis (SDS‐PAGE; 8–12% gels) and blotted onto polyvinylidene difluoride (PVDF; IPVH00010; Millipore, MA) membranes. After blocking with 5% fat‐free milk, the membranes were incubated with the following the primary antibodies: anti‐CTRP9 rabbit antibody (bs‐15085R; 1/500; Bioss, Edinburgh, Scotland, UK), anti‐Arg‐1 mouse antibody (sc‐271430; 1/500; Santa Cruz, Santa Cruz, CA), anti‐CD163 rabbit antibody (ab182422; 1/1,000; Abcam), anti‐iNOS rabbit antibody (ab15323; 1/1,000; Abcam, Cambridge, MA), anti‐CD86 rabbit antibody (13395‐1‐AP; 1/500; Wuhan Sanying, Wuhan, China), anti‐TLR4 rabbit antibody (orb106551; 1/1,000; Biorbyt, San Francisco, CA), anti‐MyD88 rabbit antibody (#4283; 1/1,000; CST, Beverly, MA), anti‐TLR4/MD2 Complex rat antibody (ab95562; 1/500; Abcam), anti‐p‐AMPKα(Thr172) rabbit antibody (#50081; 1/1,000; CST), anti‐AMPKα rabbit antibody (#5831; 1/2,000; CST), anti‐p‐NF‐κB p65 rabbit antibody (ab86299; 1/500; Abcam), anti‐NF‐κB p65 rabbit antibody (ab16502; 1/3,000; Abcam), and anti‐GAPDH rabbit antibody (ab37168; 1/10,000; Abcam). The membranes were then incubated with secondary horseradish peroxidase (HRP)‐goat antirabbit (AS1107; 1:10,000; Aspen Biotechnology, Bedford, MA) or HRP‐goat anti‐mouse (AS1106; 1:10,000; Aspen Biotechnology) at room temperature for 1 hr, and exposed to enhanced chemiluminescence (ECL) substrate (AS1027; Aspen Biotechnology). Western blot results were quantified by densitometry (Image Lab, Hercules, CA).

### 2.6 Cytokine quantification

Following treatment, IL‐1β, IL‐6, and IL‐10 levels in cell culture supernatants and heart tissue were measured using rat ELISA kits according to the manufacturer's instructions (Elabscience, Wuhan, China). Protein levels were calculated from a cytokine standard curve. Tissue ELISA measurements were normalized to the protein content of the homogenates (μg proteins/ml). All samples were analyzed in triplicate.

### 2.7 Flow cytometry

Suspended macrophages were transferred to fluorescence‐activated cell sorting (FACS) tubes. Then, cells were centrifuged and washed with 1 ml of FACS buffer. We adjusted the cell density and incubated equal numbers of cells for 20 min at 4°C with Alexa Fluor 647‐F4/80 antibody (sc‐377009 AF647; Santa Cruz) and PE (A10543; Invitrogen)‐conjugated CD163 antibody (MA5–16658; Invitrogen), followed by further incubation with PE‐conjugated iNOS (sc‐7271 PE; Santa Cruz) for 20 min. The reagents of a fixation/permeabilization Kit (GAS004; Invitrogen) were added to stained cells according to the manufacturer's instructions. Data were acquired on a FACSCalibur flow cytometer (BD Biosciences, Triangle, NC) and analyzed using FlowJo software (Tree Star, Ashland, OR). iNOS^+^/F4/80^+^ macrophages were regarded as M1 macrophages, and CD163^+^/F4/80^+^ macrophages were regarded as M2 macrophages.

### 2.8 Masson's trichrome staining

Masson's trichrome staining was performed to evaluate infarct size in heart tissue sections. Fibrosis was measured via Image‐Pro Plus (Media Cybernetics, Maryland, GA). With respect to clinical significance, only rats with large infarctions (>30%) were selected for analysis. Digitized pictures were analyzed by planimetry, and the infarcted area was measured as the percentage of stained fibrosis area/total LV area.

### 2.9 Immunofluorescence

Three ventricular sections from each rat were incubated with individual primary antibodies to CD68 (GB11067; 1/3,000; Servicebio Technology, Wuhan, China), CD163 (ab182422; 1/500; Abcam), or CD86 (13395‐1‐AP; 1/500; Wuhan Sanying). Subsequently, sections were incubated with secondary antibodies conjugated with fluorescence. The slides were washed three times with PBS, incubated in 4′,6‐diamidino‐2‐phenylindole (G1012; 1:1; Servicebio Technology) for 10 min, and then dried and coverslipped for evaluation. Fluorescent signals of 5 random nonoverlapping fields were captured with a fluorescence microscope (Nikon Eclipse C1; Nikon, Tokyo, Japan). All images were analyzed by Image‐Pro Plus 6.0 software. Quantification was performed by calculating the percentage of the positively stained area to the total area at a ×400 magnification.

### 2.10 Effects of CTRP‐9 on binding to a TLR4/MD2 fusion protein

We validated whether CTPR9 could compete with LPS for binding to TLR4/MD2‐MyD88. We simulated the process using the following steps, which were derived from an ELISA‐based assay at a wavelength of 450 nm (Brandl, Gluck, Hartmann, Salzberger & Falk, 2005; Kopp et al., 2010).

First, we designed the TLR4/MD2 molecule (Bioyeartech, Wuhan, China), which was prepared by fusing MD2 with a C‐terminal His tag to the C terminus of soluble TRL4 via a flexible linker during plasmid construction (Flag‐TLR4‐Linker‐MD2–6 × His‐pcDNA3.1 + vector; LPS trap) and transfected into macrophages. The primer sequences used are listed in Table 2. This so‐called LPS trap was successfully proven to bind LPS in RAW 264.7 cells (Brandl et al., 2005). An anti‐Flag antibody (M2; 66008‐2‐Ig; Wuhan Sanying) was coated on a plate at an appropriate dilution (2 μg/ml) in carbonate buffer and then added to the cell lysate (containing the LPS trap) and specifically bound to M2 on the ELISA plate. Second, we constructed bio‐LPS (B2643; Sigma‐Aldrich). Third, a 100‐fold dose of nonbiotinylated LPS and three different concentrations of gCTRP‐9 were added in groups to simultaneously compete with bio‐LPS for binding to the LPS trap. Then, to rule out the possibility that the effects of gCTRP9 on the LPS trap were attributable to the fact that gCTRP9 binds directly to bio‐LPS, we coated gCTRP‐9 (0 and 10 μg/ml) and then added different concentrations of bio‐LPS. Streptavidin‐HRP (SA00001‐0; Wuhan Sanying) was diluted to 0.5 μg/ml, and the reaction was terminated after adding the substrate solution to each well. Finally, to confirm that gCTRP9 effectively coated the enzyme labeling board, we coated a plate with a different concentration of gCTRP‐9 per well and recorded the fluorescence values with an ELISA‐based system after the application of an anti‐CTRP‐9 (1 μg/ml) antibody and a corresponding secondary antibody (peroxidase‐labeled antibody to rabbit immunoglobulin G (H + L; 0.2 μg/ml; 5220‐0336; seracare, Gaithersburg, MD).

**Table 2:** Information of primers for plasmid construction

Gene name	Forward sequence (5′–3′)	Reverse Sequence(5′‐3′)
TLR4	ATGATGCCTCTCTTGCATCT	CACACTGACCACCGATACACT
MD2	ATGTTGCCATTTTTTCTCTTT	ATTAACATTATGGTGGTGAAT
TLR4‐linker	CTAGTCCAGTGTGGTGGAATTCATGATTACAAGGATGACGACGATAAGATGATGCCTCTCTTGCATCT	AAAGAGAAAAAATGGCAACATGCTGCCTCCGCCTCCGCTGCCTCCGCCTCCGCTTCCTCCGCCTCCCACACTGACCACCGATACACT
MD2‐linker	AGTGTATCGGTGGTCAGTGTGGGAGGCGGAGGAAGCGGAGGCGGAGGCAGCGGAGGCGGAGGCAGCATGTTGCCATTTTTTCTCTTT	ACGGGCCCTCTAGACTCGAGTTACTAATGGTGATGGTGATGATGATTAACATTATGGTGGTGAAT

*Note*. TLR4: toll‐like receptor 4; MD2: myeloid differentiation protein 2.

### 2.11 Coimmunoprecipitation

Macrophages were treated with LPS (100 ng/ml) and 3 µg/ml gCTRP9, MD2‐neutralizing antibody (anti‐MD2; 100 ng/ml) or vehicle for 15 min. The appropriate volume of total protein extraction reagent was added to the cells. The immunoprecipitated cell extracts were incubated with an anti‐MD2 (ab24182; or anti‐TLR4 antibody 1:200; Abcam) for 1 hr, and immunoprecipitation was performed with SureBeads™ StarterKit Protein A (#1614813; Bio‐Rad, Hercules, CA) at 4℃ overnight. Treated magnetic beads were resolved by 8–12% SDS‐PAGE and transferred to PVDF membranes. Samples were immunoblotted to detect TLR4 (or MyD88) as a coprecipitated protein, and membranes were then probed with specific primary and secondary antibodies. The immune complexes were exposed in a dark box instrument after adding enhanced ECL reagent, and the AlphaEaseFC software (Kreuzbergstr, Berlin, Germany) processing system analyzed the optical density of the target band.

### 2.12 Statistical analysis

Unpaired Student's *t* tests were performed between any two groups, and multiple comparisons were evaluated by one‐way analysis of variance and Tukey's test using GraphPad Prism 5.01 (San Diego, CA). Kaplan‐Meier survival analysis was performed by using the log‐rank test. Measurement results are presented as the mean ± standard error of the mean (*SEM*). Each experiment was repeated at least three times, and data were considered to be significantly different at *p* < 0.05.

## 3 RESULTS

### 3.1 Adenoviral supplementation of CTRP9 treatment protects Day 7 post‐MI cardiac dysfunction

**Figure 1:**
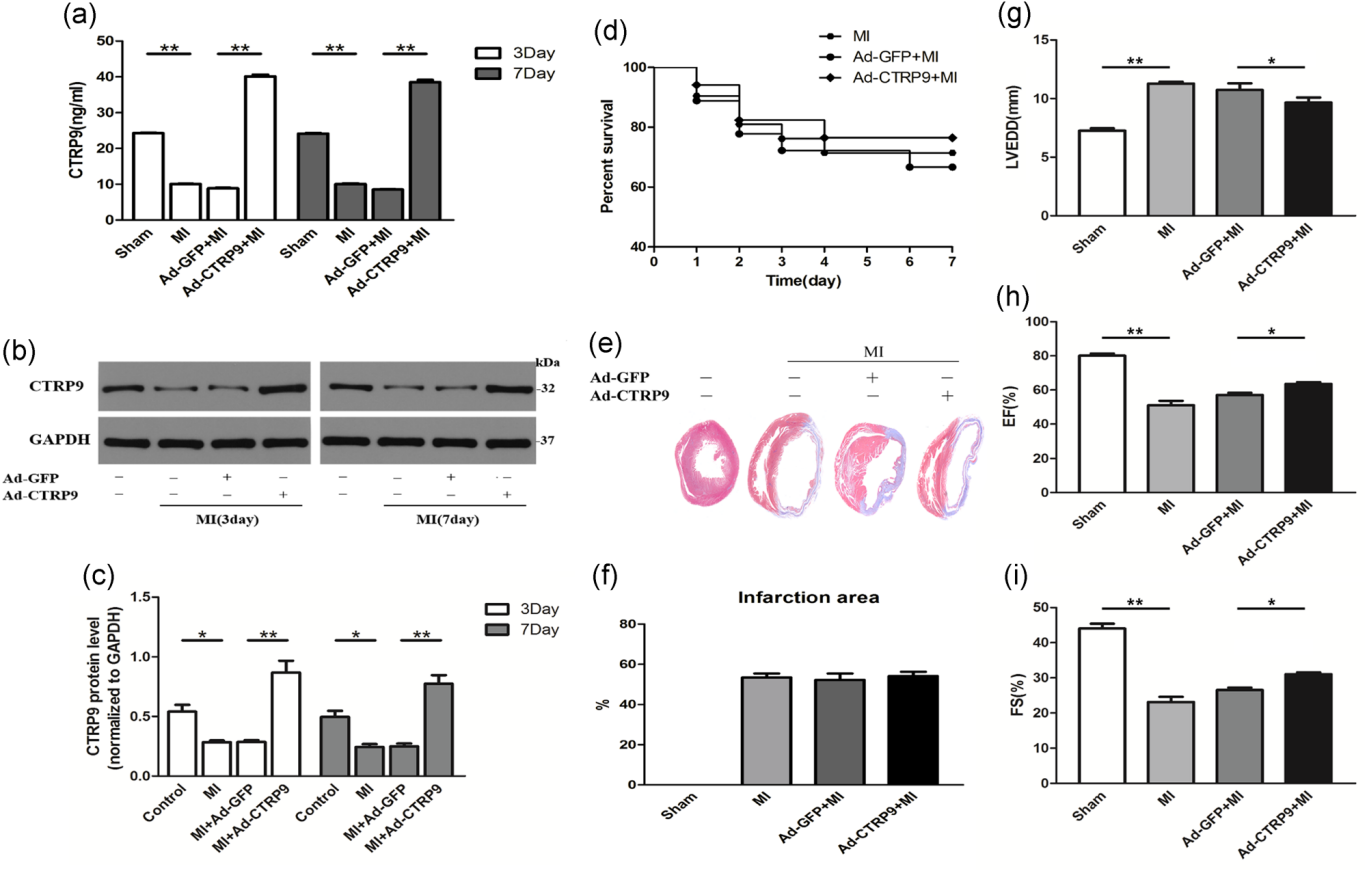
Adenoviral CTRP9 delivery ameliorates cardiac function on Day 7 post MI. Ad‐CTRP9 or Ad‐GFP was injected into the jugular vein of rats 5 days before MI. (a–c) Effects of adenoviral CTRP9 supplementation on plasma and tissue CTRP9 protein levels by ELISA and western blot analysis. (d) Survival rate according to survival curve analysis (MI, *n* = 21, Ad‐GFP, *n* = 18; Ad‐CTRP9, *n* = 17). (e,f) Cardiac Masson trichrome staining and infarct area (per group, *n* = 5). (g–i) Quantitative analysis of the LVEDD, EF, and FS of rat hearts pretreated with Ad‐CTRP9 or Ad‐GFP (per group, successively, *n* = 7, 6, 6, and 7). *p < 0.05 and **p < 0.01, the connection represents a comparison between two groups. CTRP9: C1q/TNF‐related protein‐9; EF: ejection fraction; ELISA: enzyme‐linked immunosorbent assay; FS, fractional shortening; GAPDH: glyceraldehyde 3‐phosphate dehydrogenase; GFP: green fluorescent protein; LVEDD: left ventricular end‐diastolic dimension; MI: myocardial infarction [Color figure can be viewed at wileyonlinelibrary.com]

We assessed the effect of adenoviral production of fCTRP9 on adverse ventricular dysfunction post MI. ELISA and western blot analysis showed that the protein levels of CTRP9 were significantly increased in plasma and the infarcted border area of the LV at 3 and 7 days after the injection of Ad‐CTRP9, respectively (Figure 1a–c). CTRP9 overproduction did not affect survival rates (Figure 1d), the infarct area (Figure 1e,f) or rupture rates (3:6, 50% for Ad‐GFP; and 2:4, 50% for Ad‐CTRP9) and partly restored left cardiac function (Figure 1g–i) at Day 7 post MI compared to that observed in the MI and Ad‐GFP + MI groups.

### 3.2 fCTPR9 suppresses the inflammatory response on Day 3 and stimulates M2 macrophage polarization in the infarct border zone

**Figure 2:**
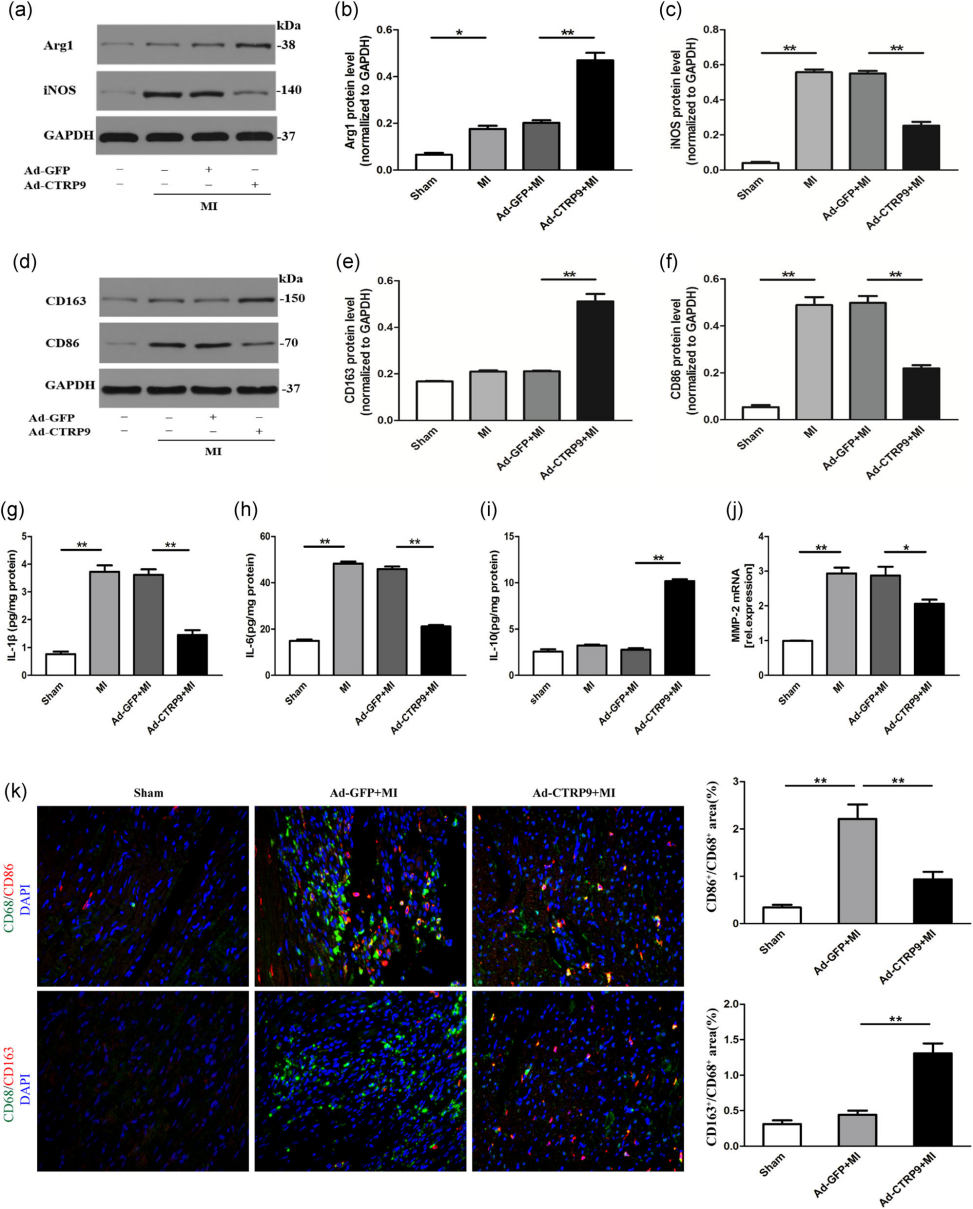
Ad‐CTRP9 treatment enhances anti‐inflammatory effects and mediates M1 to M2 phenotype polarization in the infarct border zone at Day 3 post‐MI. Rats were treated with Ad‐CTRP9 as shown in the above illustration. (a–f) The effect of Ad‐CTRP9 on M1 (iNOS and CD86) and M2 (Arg1 and CD163) macrophage markers by western blot analysis. (g–i) The protein levels of IL‐1 β, IL‐6, and IL‐10 were measured by tissue ELISA. (j) The matrix metalloproteinase (MMP)‐2 mRNA levels were measured by PCR. (k, l) Representative fluorescent immunostaining images after merging (left, *n* = 4). The hearts were stained with CD68 (green), CD86/CD163 (red), and DAPI (blue). The double‐positive CD86 and CD68 (CD86^+^/CD68^+^) areas of M1 macrophages and CD163^+^/CD68^+^ M2 macrophages were quantified (right). Data are presented as the mean ± *SEM* from three independent experiments. *p < 0.05 and **p < 0.01, the connection represents a comparison between two groups. Arg1: arginase1; CTRP9: C1q/TNF‐related protein‐9; DAPI: 4′,6‐diamidino‐2‐phenylindole; ELISA: enzyme‐linked immunosorbent assay; GAPDH: glyceraldehyde 3‐phosphate dehydrogenase; iNOS: inducible nitric oxide synthase; MI: myocardial infarction; mRNA: messenger RNA; PCR: polymerase chain reaction; *SEM*: standard error of the mean [Color figure can be viewed at wileyonlinelibrary.com]

Earlier conversion from an inflammatory to a reparative macrophage phenotype is potentially cardioprotective. The turning point for M1 to M2 macrophages is 3 days after MI localization at the border zone (Shirakawa et al., 2018). Therefore, to determine the effect of Ad‐CTRP9 on macrophage markers and inflammatory substances, tissues were detected from the infarct border zone at Day 3 post MI. As shown in Figure 2a–f, CTRP9 significantly increased the expression levels of typical M2 markers (Arg1 and CD163) and reduced the expression levels of M1 markers (iNOS and CD86) compared with MI‐induced rat in heart tissue. CTRP9 dramatically suppressed the levels of the proinflammatory cytokines IL‐1β and IL‐6 (Figure 2g,h) and upregulated the anti‐inflammatory cytokine IL‐10 (Figure 2i) in post‐MI heart tissue. We determined the effect of CTRP9 overexpression on MMP‐2 mRNA expression, and MMP‐2 mRNA levels increased in post‐MI hearts, whereas the response was blunted by fCTRP9 (Figure 2j). We identified the effect of CTRP9 on macrophage differentiation by detecting specific surface markers. Immunofluorescence staining was performed to quantify the area percentage of M1 (CD86^+^/CD68^+^) and M2 (CD163^+^/CD68^+^) macrophages in infarcted border myocardium. Immunofluorescence analysis demonstrated that CD86^+^/CD68^+^ macrophages were obviously infiltrated, and CD163^+^/CD68^+^ macrophages did not markedly increase in the Ad‐GFP + MI group on Day 3 after MI. CTRP9 treatment reduced the recruitment of M1 macrophages and elevated the expression of M2 macrophages after MI injury (Figure 2k,l).

### 3.3 CTRP9 enhances an anti‐inflammatory phenotype transition and decreases inﬂammatory factors produced by LPS + INF‐γ‐triggered macrophages

**Figure 3:**
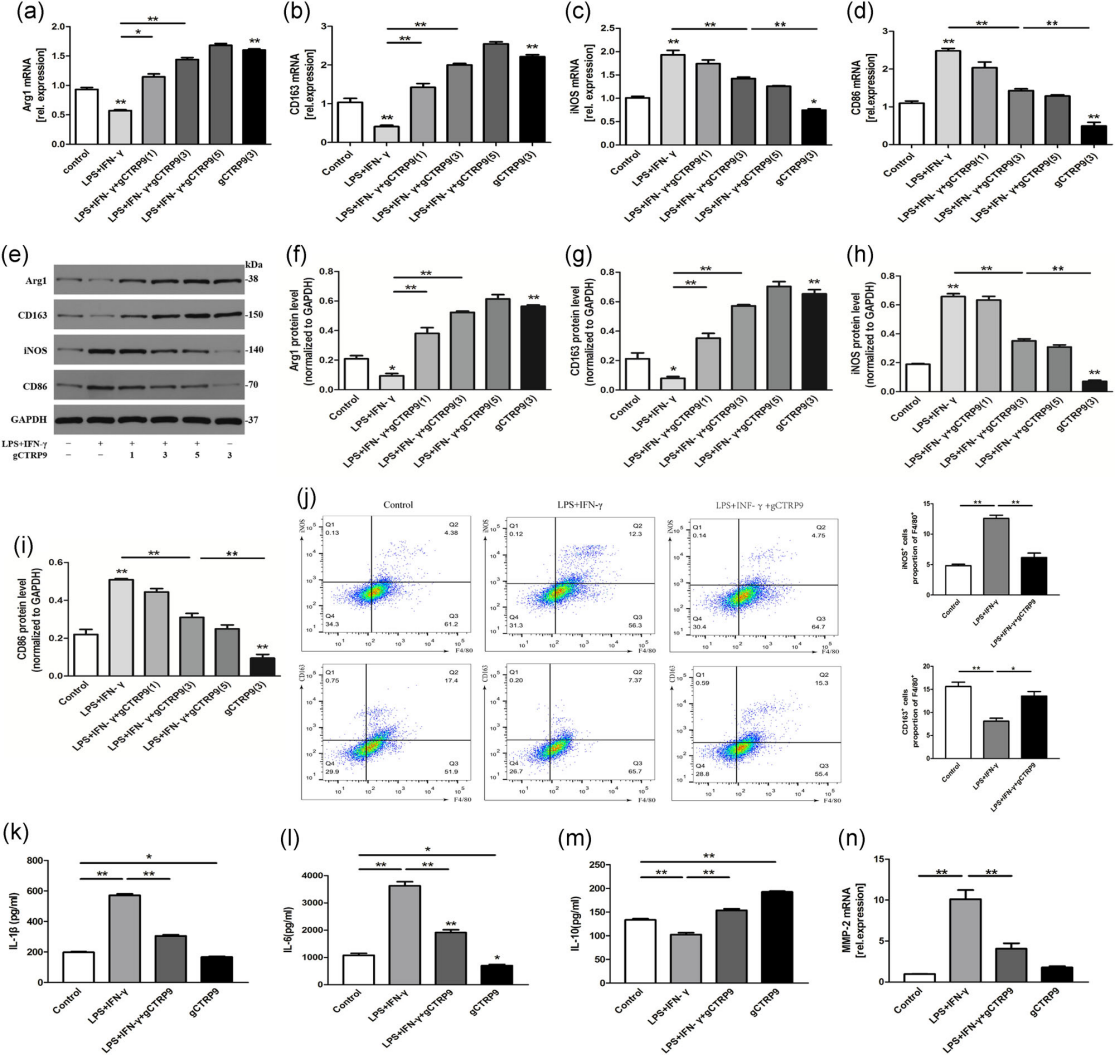
CTRP9 promotes M2 macrophage polarization and decreases inﬂammatory cytokine production in vitro. Peritoneal macrophages (1 × 10^6^ cells/ml per well) were preincubated with different concentrations of gCTRP9 (1, 3, and 5 μg/ml) for 30 min. After stimulation with 100 ng/ml LPS + 20 ng/ml IFN‐γ or treatment with PBS for 24 hr, the cells were collected. (a–i) Representative mRNA (upper panel) and protein (lower panel) expression of specific M1 and M2 markers were evaluated by qRT‐PCR and western blot analyses. (j) Flow cytometry analysis (left) with quantification (right) of iNOS^+^/F4/80^+^ and CD163^+^/F4/80^+^ macrophages in total macrophages. (k–m) The supernatant protein levels of IL‐1β, IL‐6, and IL‐10 were measured by ELISA. (n) PCR analysis was performed on mRNA for MMP‐2. At least three independent experiments were performed. Data are presented as the mean ± *SEM*. *p < 0.05 and **p < 0.01 (vs. control), the connection represents a comparison between two groups. Arg1: arginase1; CTRP9: C1q/TNF‐related protein‐9; ELISA: enzyme‐linked immunosorbent assay; GAPDH: glyceraldehyde 3‐phosphate dehydrogenase; IFN‐γ: interferon‐γ; iNOS: inducible nitric oxide synthase; LPS: lipopolysaccharide; mRNA: messenger RNA; PCR: polymerase chain reaction; qRT‐PCR: quantitative reverse‐transcription PCR [Color figure can be viewed at wileyonlinelibrary.com]

To investigate the phenotypic character of macrophages in the presence of gCTRP9 in vitro, we collected peritoneal macrophages from SD rats. We measured the mRNA and protein expression of M1 (iNOS and CD86) and M2 (Arg1 and CD163) markers in macrophages from macrophages treated with LPS + INF‐γ and/or different concentrations of gCTRP9. The M1 markers significantly increased and M2 markers moderately decreased above basal levels at 24 hr after LPS + INF‐γ stimulation, and gCTRP9 significantly elevated M2 markers and reduced M1 markers in a concentration‐dependent manner. The results showed that the effect of gCTRP9 comprehensively began at a concentration of 3 μg/ml, and the expression of M2 instead of M1 marker was improved at 1 μg/ml, which differed from the 1 μg/ml inhibitory concentration of CTRP9 in the oxidized low‐density lipoprotein‐induced inflammatory reaction (Zhang et al., 2016); therefore, we adopted the concentration of 3 μg/ml in the following experiments. gCTRP9 had a similar effect on macrophage polarization in the absence of LPS IFN‐γ intervention (Figure 3a‐i). We also observed the elevation of this phenomenon, in which the percentage of M1‐like macrophages was lower while M2‐like macrophages were increased, upon cotreatment of lipopolysaccharide + interferon‐γ (LPS + IFN‐γ) with gCTRP9 by flow cytometry analysis (Figure 3j).

To evaluate the effects of gCTRP9 on the release of proinflammatory cytokines, the supernatant protein levels of IL‐1β, IL‐6, and IL‐10 were determined by ELISA kits. As shown in Figure 3k–m, LPS + INF‐γ increased the levels of IL‐6 and IL‐1β and decreased the levels of IL‐10, whereas gCTRP9 effectively reversed these changes. Inflammatory treatment of peritoneal macrophages with LPS + INF‐γ resulted in robust transcription of MMP‐2, and gCTRP9 treatment resulted in the secretion of lower levels of MMP‐2 (Figure 3n).

### 3.4 CTRP9 deficiency increases the M1 macrophage phenotype at baseline and among LPS + IFN‐γ‐induced macrophages

**Figure 4:**
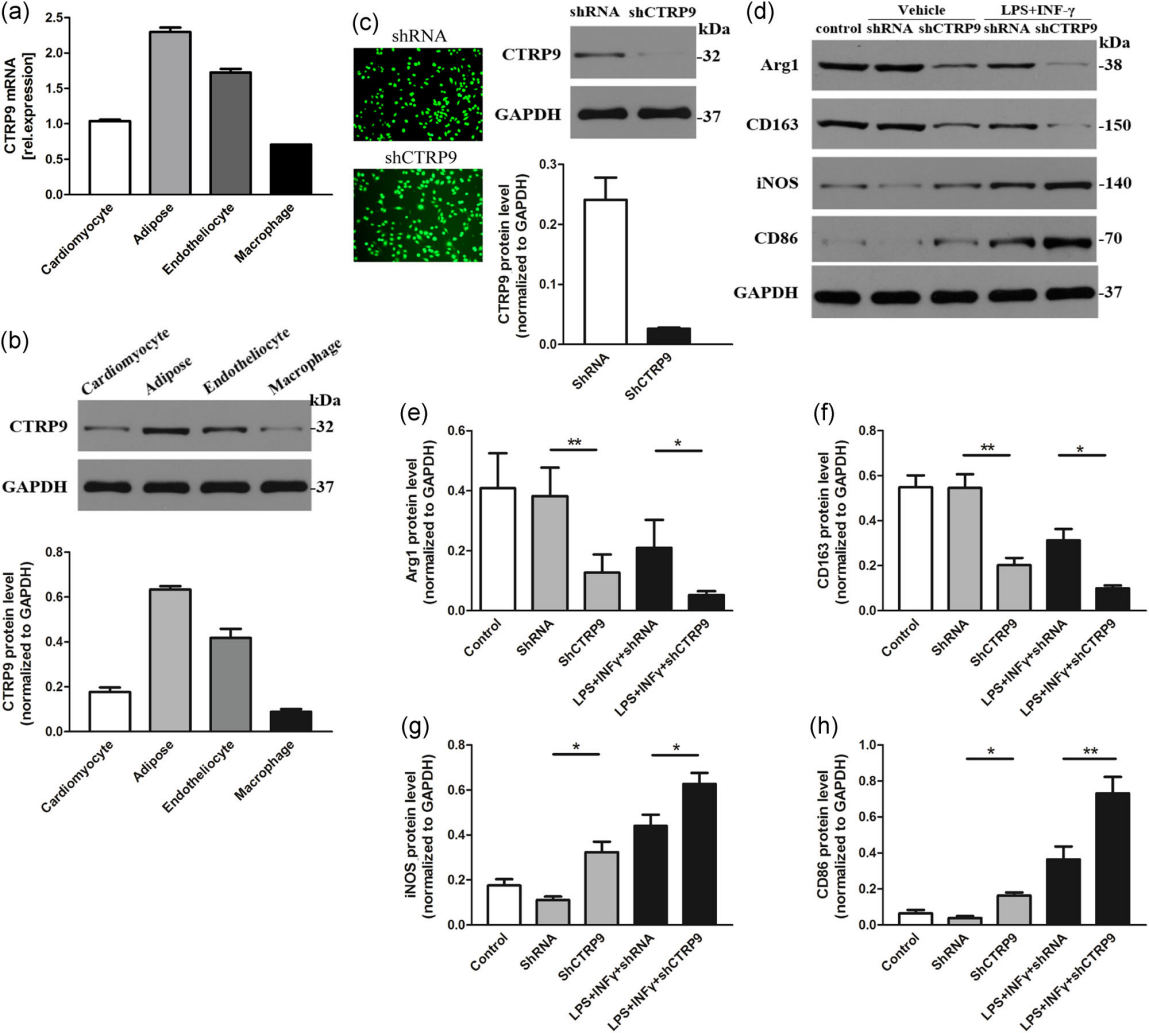
CTRP9 deficiency enhances the M1 macrophage phenotype. (a–b) mRNA and protein levels of CTRP9 in 4 kinds of cells. (c) Representative fluorescence microscopy images of GFP fluorescence in macrophages after transfection of shRNA and shCTRP9, and corresponding protein levels determined by western blot analysis. (d–h) Protein levels of M1 and M2 macrophage markers after knockdown of CTRP9 at baseline and in LPS + IFN‐γ‐induced macrophages for 24 hr. Data are presented as the mean ± *SEM* from four independent experiments. *p < 0.05 and **p < 0.01, the connection represents a comparison between two groups. Arg1: arginase1; GAPDH: glyceraldehyde 3‐phosphate dehydrogenase; GFP: green fluorescent protein; IFN‐γ: interferon‐γ; LPS: lipopolysaccharide; mRNA: messenger RNA; *SEM*: standard error of the mean; shRNA: small hairpin RNA [Color figure can be viewed at wileyonlinelibrary.com]

Next, we investigated whether reduced CTRP9 levels affect macrophage phenotype. First, we detected the mRNA and protein expression of CTRP9 in four types of cells (Figure 4a,b). Subsequently, we knocked down CTRP9 in macrophage cells to verify the effect of targeted knockdown by shRNA, and we evaluated the efficiency of this approach by western blot (Figure 4c). shRNA had no significantly distinctive effect on cells compared to that of the control group. Downregulation of CTRP9 increased the protein expression levels of iNOS and CD86, and decreased the protein expression levels of Arg1 and CD163 production under basal conditions, whereas silencing CTRP9 more significantly worsened the above state compared to treatment with LPS + IFN‐γ + (Figure 4d–h). This evidence suggests that the absence of CTRP9 enhances the conversion of M1 macrophages.

### 3.5 CTRP9 shows a similar function to IL‐4 by changing the polarization state of M1 and enhancing anti‐inflammatory responses

**Figure 5:**
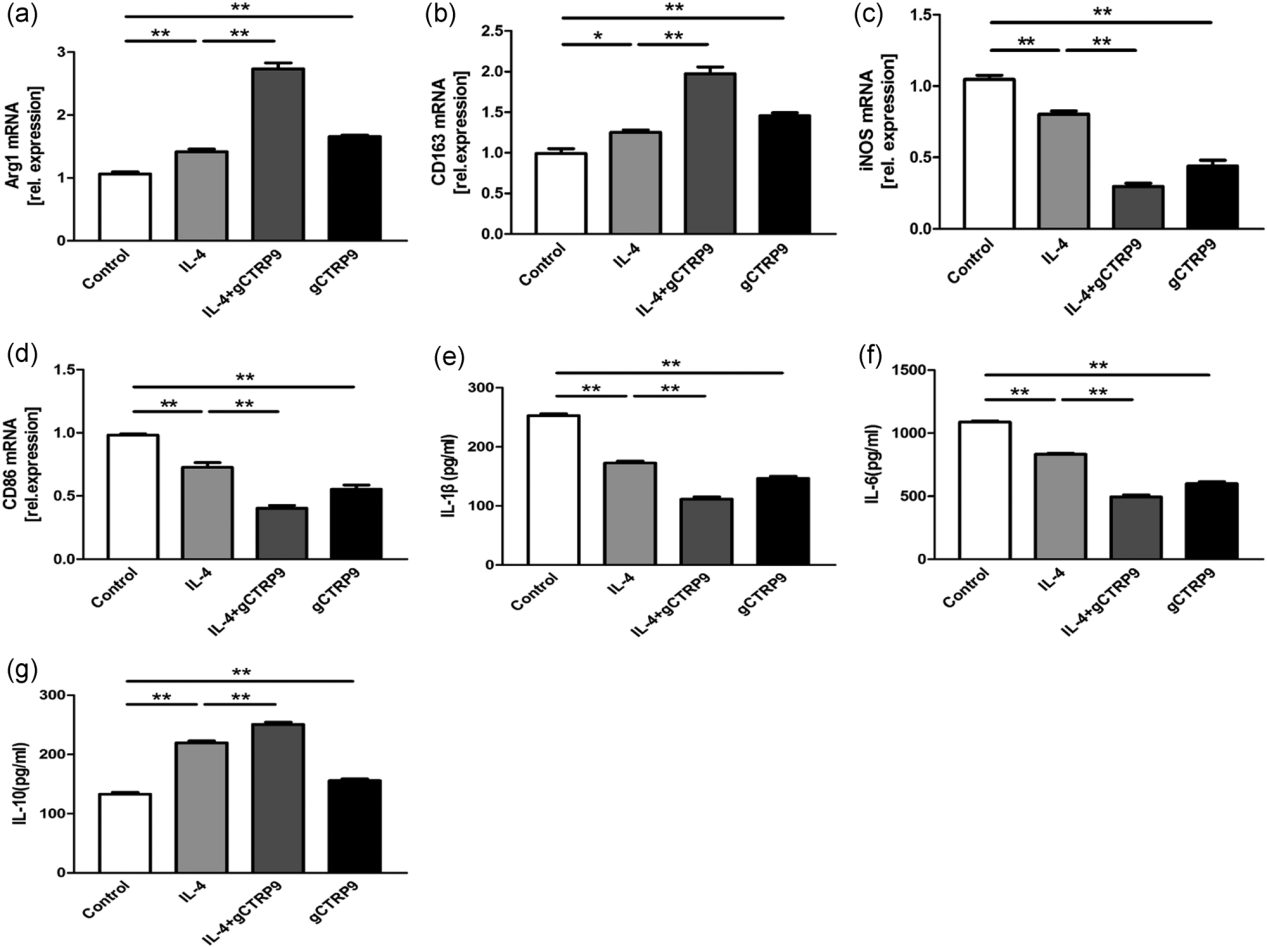
CTRP9 has a similar function to that of IL‐4 and promotes an anti‐inflammatory macrophage phenotype. (a–d) mRNA levels of specific M1 and M2 markers under treatment with gCTRP9 (3 μg/ml), IL‐4(20 ng/ml), or both by RT‐PCR. (e–g) The supernatant protein levels of IL‐1β, IL‐6, and IL‐10 were measured by ELISA. Data are shown as the mean ± *SEM* from three independent experiments. *p < 0.05 and **p < 0.01, the connection represents a comparison between two groups. Arg1: arginase1; CTRP9: C1q/TNF‐related protein‐9; ELISA: enzyme‐linked immunosorbent assay; IL: interleukin; mRNA: messenger RNA; RT‐PCR: reverse‐transcription polymerase chain reaction; *SEM*: standard error of the mean

To demonstrate whether gCTRP9 function resembled the function of IL‐4, which typically acts by promoting M2 macrophage phenotype transition, treatment of peritoneal macrophages with gCTRP9 for 24 hr led to the upregulated expression of the biomarkers Arg1 and CD163 and the downregulation of iNOS and CD86 at the mRNA level. The magnitude of marker variation in the presence of gCTRP9 was similar to that seen with IL‐4, a prototypical Th2 cytokine which has been thought to promote macrophage M1/M2 polarization. Cotreatment with gCTRP9 and IL‐4 changed the mRNA levels of these markers in an additive manner (Figure 5a–d). Meanwhile, treatment with gCTRP9, IL‐4, or both dramatically diminished the levels of the proinflammatory factors IL‐1β and IL‐6 and increased the release of IL‐10 (Figure 5e–g). Therefore, we deem that CTRP9 has a similar effect as IL‐4 in terms of motivating M2 polarization and anti‐inflammatory ability, rather than decreasing function.

### 3.6 CTRP9 promotes macrophage polarization partly through AMPK‐mediated activation and largely via an NF‐κB‐dependent pathway

**Figure 6:**
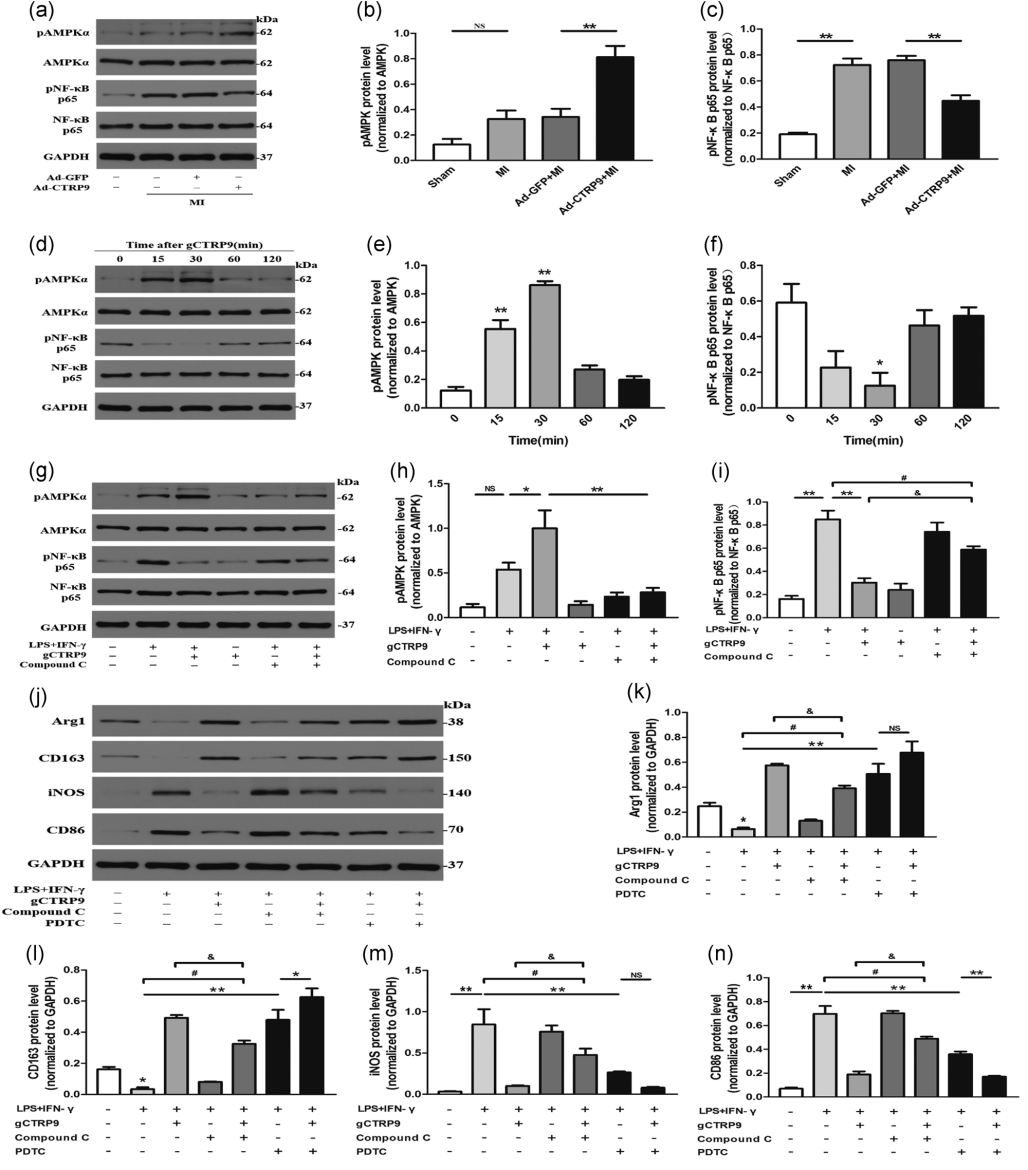
CTRP9 promotes macrophage polarization partly through AMPK‐mediated activation and largely via an NF‐κB‐dependent pathway. (a–c) The effects of Ad‐GFP and Ad‐CTRP9 transfection on the phosphorylation of AMPK and NF‐κB p65 in the infarct border zone at Day 3 post MI by western blot. (d–f) Effects of treatment of macrophages with gCTRP9 (3 μg/ml) at different time points on AMPK (Thr172) and NF‐κB p65 phosphorylation by western blot analysis. (g–i) Western blot analysis assessed the effects of the AMPK inhibitor compound C on pAMPKα and pNF‐κB p65 protein expression in macrophages responding to gCTRP9 by LPS + IFN‐γ stimulation. Cells were treated with compound C (10 mol/l) for 2 hr and then incubated with CTRP9 (3 µg/ml) for 30 min, followed by LPS + IFN‐γ challenge for 24 hr. (j–n) Effects of two inhibitors (compound C or PDTC; 100 μM) on M1 and M2 marker protein expression in macrophages in response to gCTRP9 via LPS + IFN‐γ stimulation using western blot analysis. Data are shown as the mean ± *SEM* from three independent experiments; *p < 0.05, **p < 0.01 (vs. control group), ^&,#^ *p* < 0.05, and NS = *p* > 0.05, the connection represents a comparison between two groups. AMPK: adenosine monophosphate kinase; Arg1: arginase1; CTRP9: C1q/TNF‐related protein‐9; GAPDH: glyceraldehyde 3‐phosphate dehydrogenase; GFP: green fluorescent protein; IFN‐γ: interferon‐γ; LPS: lipopolysaccharide; MI: myocardial infarction; NF‐κB: nuclear factor‐κB; NS: not significant; PDTC: pyrrolidine dithiocarbamate; *SEM*: standard error of the mean

**Figure 7:**
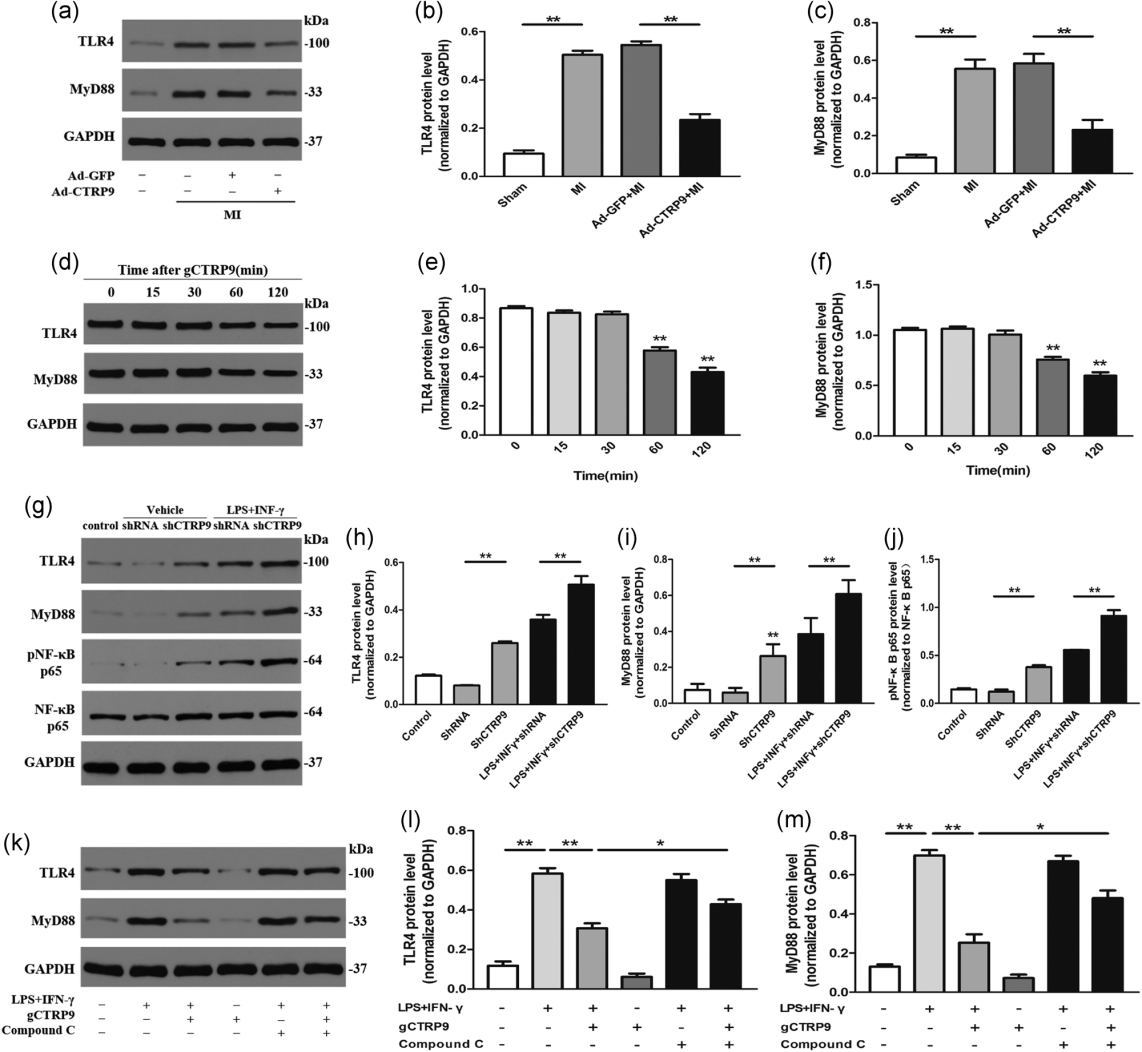
CTRP9‐mediated downregulation of TLR4‐MyD88 protein expression is associated with AMPK phosphorylation. (a–c) TLR4 and MyD88 protein expression in the infarct border zone at Day 3 post MI with Ad‐GFP and Ad‐CTRP9 pretreatment. (d–f) Effects of TLR4 and MyD88 on macrophages treated with gCTRP9 (3 μg/ml) at different time points by western blot analysis. (g–j) Protein levels of TLR4 and MyD88 after knockdown of CTRP9 in macrophages for 24 hr. (k–m) Effects of compound C on TLR4 and MyD88 protein expression in macrophages responding to gCTRP9 via LPS + IFN‐γ stimulation using western blot analysis. Data are presented as the mean ± *SEM* from four independent experiments; *p < 0.05 and **p < 0.01, the connection represents a comparison between two groups. AMPK: adenosine monophosphate kinase; CTRP9: C1q/TNF‐related protein‐9; GAPDH: glyceraldehyde 3‐phosphate dehydrogenase; GFP: green fluorescent protein; IFN‐γ: interferon‐γ; LPS: lipopolysaccharide; MI: myocardial infarction; MyD88: myeloid differentiation factor 88; NF‐κB: nuclear factor‐κB; SEM: standard error of the mean; shRNA: small hairpin RNA; TLR4: toll‐like receptor 4

As summarized in Figure 6, Ad‐CTRP9 replenishment significantly activated AMPK and inhibited NF‐κB p65 phosphorylation in a post‐MI model (Figure 6a–c). To obtain more reliable evidence, we utilized peritoneal macrophages for an in vitro experiment in which gCTRP9‐induced AMPK phosphorylation (at Thr172 of its α subunit; Figure 6d,e) and NF‐κB p65 dephosphorylation (Figure 6d,f) at 3 μg/ml was observed within 30 min of gCTRP9 administration in a time‐dependent manner, followed by reduction to approximately normal levels with time; the chronergy was analogous to the effect of CTRP9 in cardiomyocytes (Zhang et al., 2016). We further assessed the role of gCTRP9 in LPS + INF‐γ‐induced AMPK and NF‐κB p65 phosphorylation expression in macrophages by western blot, and we found that gCTRP9 promoted AMPK phosphorylation (Figure 6g,h) and attenuated NF‐κB p65 phosphorylation (Figure 6g,i). We also observed that silencing CTRP9 led to increased p‐NF‐κB p65 in macrophages (Figure 7j). Simultaneously, we treated macrophages with the specific AMPK inhibitor compound C to further evaluate the effect of CTRP9 on AMPK and the effect of AMPK on NF‐κB phosphorylation under LPS + INF‐γ stimulation. The compound C strongly inhibited p‐AMPKα and motivated p‐NF‐κB p65. However, we saw that use of compound C partially offset the inhibition of p‐NF‐κB p65 by gCTRP9 (Figure 6g,i; Label: &) but still did not restore p‐NF‐κB p65 levels with LPS + IFN‐γ stimulation (Figure 6g,i; Label: #), so we considered whether gCTRP9 had another mechanism to inhibit NF‐κB p65 phosphorylation.

To determine the mechanisms by which gCTRP9 induces macrophage polarization transition via AMPK and NF‐κB signaling, we used compound C or the NF‐κB inhibitor PDTC to interfere with the efficacy of gCTRP9 for the M1/M2 markers by LPS + IFN‐γ stimulation. Most importantly, pretreatment with compound C partly (Figure 6j–n; Label: &) but not completely (Figure 6j–n; Label: #) abolished the effect of CTRP9 on the promotion of M2 markers and suppression of M1 marker transition in the case of LPS + IFN‐γ stimulation. The efficiency of PDTC antagonization LPS + INF‐γ‐induced marker changes was similar to that of gCTRP9. Moreover, the addition of gCTRP9 increased the effect of PDTC on augmented CD163 generation, weakened CD86 expression after LPSS + INF‐γ stimulation, and had no significant effect on the expression of Arg1 and iNOS (Figure 6j–n). These data suggest that NF‐κB dephosphorylation signaling plays a more significant role than AMPK phosphorylation in CTRP9‐mediated macrophage polarization. In addition to promoting AMPK phosphorylation, it is unclear whether other existing signaling systems contribute to the CTRP9‐mediated suppression of NF‐κB phosphorylation and played a role in the macrophage polarization.

### 3.7 CTRP9 reduces TLR4‐MyD88 protein expression in macrophages through AMPK activation in vitro and in vivo

To determine whether CTRP9 exerted pharmacological functions on TLR4 and MyD88 production in an AMPK‐dependent manner as a reason for p‐NF‐κB p65 downregulation, we further conducted experiments in vivo and in vitro.

As shown in Figure 7a–c, Ad‐CTRP9 markedly inhibited the overexpression of TLR4 and MyD88 at the protein level in the infarct border zone at Day 3 post‐MI. In vitro, CTRP9 inhibited the expression of TLR4 and MyD88 in macrophages in a time‐dependent manner (Figure 7d–f), and we observed that the expression of both began to decrease after 1 hr of CTRP9 intervention. Deletion of CTRP9 elevated the protein expression levels of TLR4 and MyD88 (Figure 7g–i). Compared to the LPS + IFN‐γ group, the group treated with gCTRP9 displayed obvious decreases in TLR4 and MyD88 expression, but cotreatment with compound C reversed the effects of gCTRP9 on the overexpression of TLR4 and MyD88 induced by LPS + IFN‐γ stimulation at the protein level. Additionally, compound C itself showed no effect on TLR4 and MyD88 protein levels (Figure 7k–m). These outcomes proved that antagonizing pAMPK can partly block the effects of gCTRP9 on decreased TLR4 and MyD88 in LPS + IFN‐γ‐induced macrophages.

### 3.8 CTRP9 alleviates MD2/TLR4‐MyD88 pathway activation by inhibiting the binding of TLR4 and MD2

**Figure 8:**
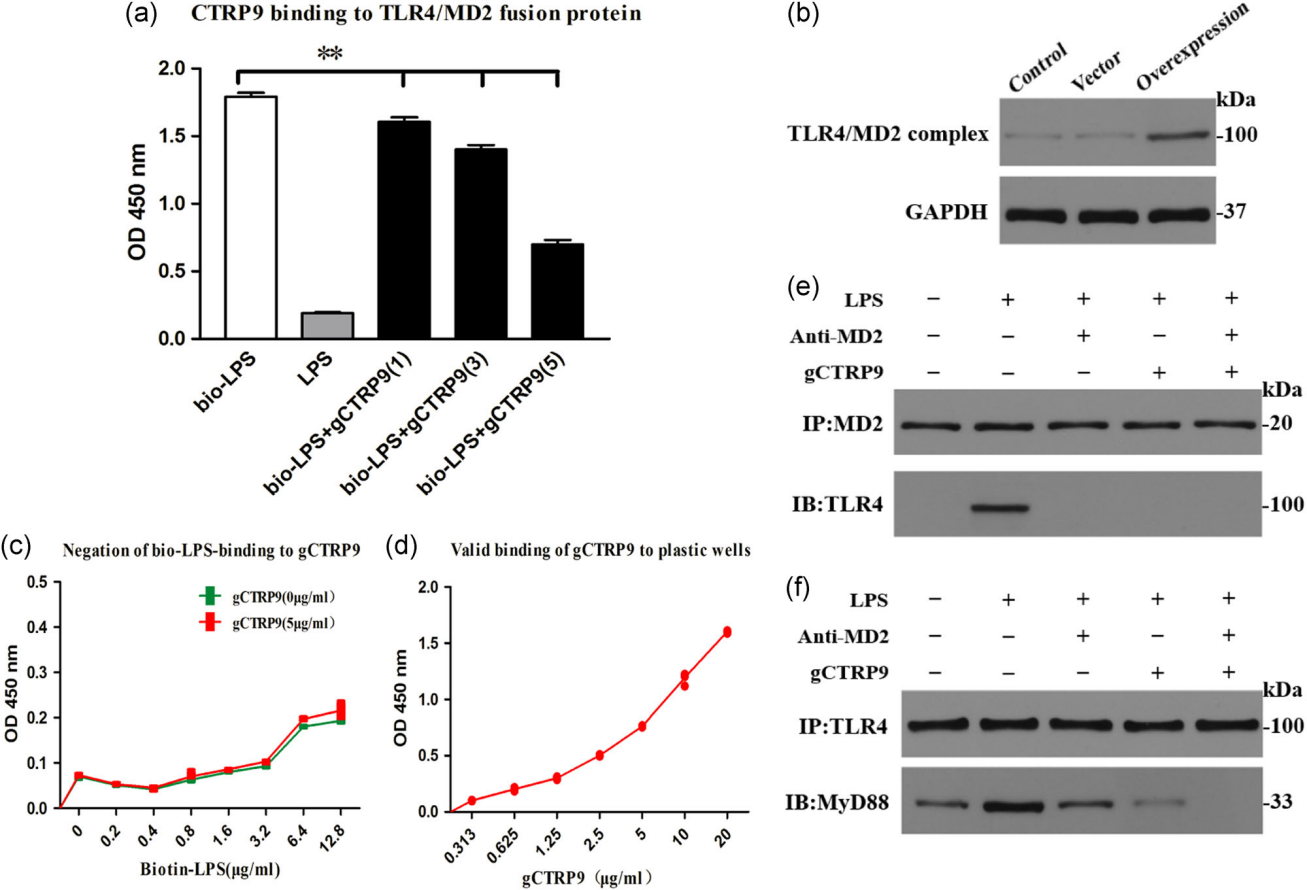
gCTRP9 inhibits TLR4/MD2 complex formation. (a) The competitive binding of bio‐LPS and gCTRP9 to the designed LPS trap was studied by using an ELISA‐based assay. The bio‐LPS (1 μg/ml) and LPS (100‐fold dose) emulously combined with the LPS trap (lanes 1 and 2). The ability of three doses (μg/ml) of CTRP9 to competitively bind bio‐LPS to the LPS trap was tested (lanes 3–5). (b) The construction of TLR4 and MD2 fusion proteins was confirmed by western blot analysis. (c) ELISA‐based analysis of the ability of bio‐LPS to bind to gCTRP9. (d) Verification of the ability of gCTRP9 to effectively bind to the plastic plate in the ELISA system. (e, f) Representative western blot (IB) from a coprecipitation (IP) experiment showing the effects of CTRP9 and anti‐MD2 on MD2/TLR4 complex formation (e) and TLR4/MyD88 complex formation (f). Data are presented as the mean ± *SEM* from three independent experiments, **p < 0.01; the connection represents a comparison between the two groups. CTRP9: C1q/TNF‐related protein‐9; ELISA: enzyme‐linked immunosorbent assay; LPS: lipopolysaccharide; MyD88: myeloid differentiation factor 88; *SEM*: standard error of the mean; TLR4: toll‐like receptor 4 [Color figure can be viewed at wileyonlinelibrary.com]

To further elucidate the role of gCTRP9 in the MD2/TLR4‐MyD88 signaling pathway, we constructed a TLR4/MD2 fusion protein in a plasmid and verified that TLR4 was successfully connected with MD2 via a linker (Figure 8b). To investigate whether CTRP9 antagonizes the binding of LPS to the LPS trap, we designed the bio‐LPS as a positive control group, which was found to bind to the LPS trap by ELISA, and this effect was reversed by non‐bio‐LPS. We then cotreated with three various concentrations of CTRP9 and bio‐LPS and observed that all three concentrations (1, 3, and 5 μg/ml) of gCTRP9 effectively suppressed bio‐LPS binding to the LPS trap in a concentration‐dependent manner (Figure 8a). We excluded the possibility that CTRP9 directly bound to bio‐LPS, and variances in values were mainly attributable to the changes in bio‐LPS concentration (Figure 8c). The colorimetric response of the corresponding secondary antibody at OD450 was consistent with the concentration of gCTRP9, and we confirmed that gCTRP9 was validly bound to plastic wells (Figure 8d).

We assessed whether gCTRP9 prevented the MD2/TLR4 complex formation and MyD88 recruitment in macrophages by immunoprecipitation. We found that LPS stimulated an intensive connection between MD2 and TLR4 (Figure 8e) and recruitment of MyD88 to the complex (Figure 8f). Nonetheless, pretreatment with an MD2‐neutralizing antibody (anti‐MD2) or gCTRP9 suppressed MD2/TLR4 complex formation (Figure 8e) and MyD88 recruitment (Figure 8f), and cotreatment of gCTRP9 and anti‐MD2 had a stronger inhibitory effect than either alone. CTRP9 appears to be essential for suppressing the TLR4 inflammatory pathway by hampering the binding of TLR4 and MD2.

## 4 DISCUSSION

In the present study, we focused on the role of the presence of CTRP9 improving cardiac function through macrophage polarization in the early stage of MI. Our results affirmed that CTRP9 in vivo attenuated post‐MI cardiac dysfunction and was dispensable for M1 to M2 macrophage polarization transition, which was further proven in the in vitro experiments. Then, we identified that these effects of CTRP9 on macrophages were regulated mainly by depressing NF‐κB p65 phosphorylation, which is involved in inhibiting the TLR4/MD2/MyD88 pathway and elevating AMPK phosphorylation.

We know that inappropriate adjustment of macrophage phenotypes and extended inflammation can impair proper tissue remodeling after MI. Hence, the early resolution of the inflammatory reactions is a pivotal juncture of cardiac remodeling. Currently, the modulation of macrophage polarization is a potential therapeutic object for the treatment of MI. CTRP9 is a well‐known adipokine that can suppress production of proinflammatory cytokines and has protective functions in the heart (Kambara et al., 2015; Sun et al., 2013; Zhang et al., 2016). Whether CTRP9 mediated macrophage polarization and specific regulatory mechanisms that directed macrophages into various functional subsets need further study to explain the protective effects of CTRP9 against anti‐inflammatory activities in the MI model of the disease.

To elucidate the effects of CTRP9 in the post‐MI heart in vivo, overexpressing CTRP9 post MI alleviated adverse cardiac dysfunction without improving the 7‐day post‐MI infarct area, rupture rates or survival rates. We can see that CTRP9 does not ameliorate early prognosis; existing research showed that exogenous CTRP9 improved the survival rate at 42 days post MI but did not decrease mortality at 7 days post MI (Sun et al., 2013). The effect of CTRP on prognosis in the early and late stages is inconsistent, which may be related to the profibrosis of M2 macrophages in the early stage of MI (Jung et al., 2017). MMP‐2, which is notably secreted by M1 macrophages and alters the extracellular matrix, participates in cardiac remodeling and boosts cardiac rupture (Sica & Mantovani, 2012). Lower MMP‐2 expression in vivo and in vitro was observed with CTPR9 pretreatment in the present study, which may have contributed to the better post‐MI contractile function but did not reduce post‐MI heart rupture. An in vivo study showed that CTRP9 obviously promoted the M1 to M2 macrophage transition, suggesting that the partial recovery of the LV function may be related to not only the regulation of inflammation via M1 macrophage inhibition but also the promotion of the M2 macrophage population.

We used peritoneal macrophages and induced them to differentiate into M1 macrophages. Conventionally, M1 macrophages were induced when macrophages were exposed to LPS + IFN‐γ, and then we conducted a series of cell studies. M1 macrophages expressed high levels of M1 markers and diminished the expression of M2 markers, which was consistent with previous research (Lin et al., 2017; Mo et al., 2014), whereas pretreatment with gCTRP9 significantly reversed these effects and was further improved by CTRP9 deficiency and the Th2 cytokine IL‐4. The anti‐inflammatory effects of CTRP 9 on inflammatory factors have also been confirmed. CTRP9 alone, in the absence of stimulators, had relatively homologous effects. Similarly, APN, a cytokine in the same family as CTRP9, also played a direct role in lung macrophage polarization (Ohashi et al., 2010). We examined the expression of CTRP9 in macrophages and found that expression in macrophages was lower than the abundant expression on cardiomyocytes, adipocytes, and endotheliocytes (Appari et al., 2017; Su et al., 2013). Taken together, our results suggested the positive regulatory function of CTRP9 in macrophage polarization and anti‐inflammatory reactions.

AMPK is not only a cellular stress sensor but also plays an important role in the suppression of inflammatory responses, and phosphorylation of the threonine‐172 residue of the α subunit is critical for AMPK activity (Steinberg & Schertzer, 2014). AMPK can attenuate inflammatory responses by suppressing the activation of NF‐κB (Yi et al., 2011). AMPK and NF‐κB p65 are involved in macrophage polarization and were both identified as effector molecules of CTRP9, as activation of AMPK exerts some potential protective functions in an MI disease model (Gu et al., 2018; J. Wang et al., 2013). In the present study, fCTPR9 increased AMPK activation and suppressed NF‐kB activation at Day 3 post‐MI in vivo. We further found that administration of CTRP9 to macrophages led to AMPK phosphorylation and NF‐κB dephosphorylation in the absence or presence of LPS + IFN‐γ in an in vitro experiment, and the time‐dependent effects were similar to those in previous studies exploring different stimulators and cells (Kambara et al., 2015; Sun et al., 2013; Zhang et al., 2016), which was further confirmed by the application of compound C and PDTC. As expected, we obtained similar results. Previous research deemed that CTRP9 can activate AMPK after MI, but CTRP9 attenuates adverse post‐MI cardiac remodeling, largely via a PKA‐dependent pathway (Sun et al., 2013); this previous study utilized H9C2 and AMPK‐DN cardiomyocytes and mainly studied apoptosis and fibrosis mechanisms after long‐term MI, whereas in this study, we mainly studied the macrophage polarization process mediated by CTRP9 in the context of early inflammatory transformation post MI. Indeed, CTRP9 protected against acute heart injury after ischemia‐reperfusion through an AMPK‐dependent mechanism (Kambara et al., 2012). Furthermore, inflammatory lesions in the early stage of myocardial infarction had a greater effect on cardiac remodeling than fibrotic processes (Prabhu & Frangogiannis, 2016). Thus, we presumed that the macrophage polarization‐promoting effect of CTRP9 may contribute to early cardioprotective function post MI by partially regulating AMPK signaling pathways. Interestingly, the extent of LPS‐induced NF‐κB p65 phosphorylation and the changes in M1 and M2 markers under CTRP9 intervention were not fully restored by preconditioning with compound C; these data suggested that other signaling pathways besides AMPK activation participate in the NF‐κB phosphorylation suppression process mediated by CTRP9, thereby affecting macrophage phenotype transformations.

The MD2/TLR4 complex acts as an upstream signaling molecule of NF‐κB, and thus, the LPS receptor is brought to our attention. MD2 is an essential extracellular molecule for LPS recognition. The binding of LPS with MD2 leads to the recruitment of the adapter protein MyD88 and the production of pNF‐κb (Y. Yang et al., 2016). CTRP9 can restrain the cholesterol‐induced activation of the TLR4 signaling pathway in VSMCs (Q. Liu et al., 2017). However, the precise molecular mechanisms remain unclear. Combined with the important role of the TLR/MyD88 downstream molecules IRF5, AP1, and NF‐κB in M1 macrophage polarization, we deduced that CTRP9 also affected macrophage polarization to participate in post‐MI protective effects via the TLR4/NF‐κB pathway.

The two key results were as follows: (a) Negative regulation of TLR4 and MyD88 protein expression. Activation of TLR4 is the pivotal process in LV remodeling following MI, and TLR4 defects and reduction of TLR4 ameliorate LV remodeling (Sheng et al., 2009; Timmers et al., 2008). Thus, in this experiment, CTRP9 reduced the expression of TLR4 and MyD88 after MI and also contributed to anti‐ventricular remodeling, which was one of the reasons for the improvement of post‐MI cardiac function. Previous research studies showed that reducing the expression of TLR4 in macrophages can decrease p‐NF‐κB, attenuate inflammatory responses and promote M1 toward M2 macrophage polarization (Sheng et al., 2009; C. Zhang et al., 2018). It has been reported that TLR‐mediated proinflammatory pathways, particularly NF‐κB, are correlated with the anti‐inflammation role of AMPK and activation of AMPK inhibits TLR4 expression (Tao et al., 2017; Y. Yang et al., 2016; C. Zhang et al., 2018). LPS + INF‐γ stimulation elevated the expression of TLR4 and MyD88, and similar results were obtained in this study, but AMPK phosphorylation increased, resulting in similar or inconsistent reports (Tao et al., 2017; C. Zhang et al., 2018). Our results indicated that AMPK was activated by CTRP9 and subsequently suppressed the TLR4 and MyD88 activity of macrophages in the presence or absence of an inflammatory stimulus and in the adaptive response to hypoxic and ischemic stress of the post‐MI heart. (b) CTRP9 directly bound to the MD2/TLR4 complex. We discuss the possibility of other mechanisms through which CTRP9 may affect p‐NF‐κB via TLR4. More studies have confirmed that certain substances and components can inhibit the downstream pathways by directly binding to the extracellular structure of TLR receptors (Kopp et al., 2010; M. Wang et al., 2011). That being the case, can CTRP9 also play a corresponding role by combining with the extracellular structure of TLR4? Our evidence supported a mechanism by which CTRP9 directly binds to MD2 to activate TLR4 signaling (MyD88‐dependent pathway), based on our construction of a TLR4/MD2 fusion protein for ELISA and immunoprecipitation analyses. Therefore, we confirmed that CTRP9 acts as a novel and endogenous LPS antagonist that directly binds to MD2 and impedes MD2/TLR4 complex formation and MyD88 recruitment. We suggest that this mechanism supplements the inhibition of NF‐κB phosphorylation by CTRP9. Competing with MD2 for TLR4 binding can block inflammatory responses (O'Reilly & van Laar, 2018), which represents a promising drug target for the treatment of inflammation‐associated diseases.

In summary, the present study established a better understanding of the protective role of CTRP9 in the early‐stage post‐MI rat. Our findings indicated that the effects associated with macrophage polarization were likely regulated by AMPK and direct binding with the MD2/TLR4 complex to influence the phosphorylation of the downstream molecule NF‐κB. The findings represent new mechanistic insights into MI and the immunity of cardiovascular diseases.

## CONFLICT OF INTERESTS

The authors declare that there are no conflict of interests.
